# Dealing with the positive publication bias: Why you should really publish your negative results

**DOI:** 10.11613/BM.2017.030201

**Published:** 2017-10-15

**Authors:** Ana Mlinarić, Martina Horvat, Vesna Šupak Smolčić

**Affiliations:** 1 Research Integrity Editor, *Biochemia Medica*; 2 Department of Laboratory Diagnostics, University Hospital Centre Zagreb, Zagreb; 3 Department of Medical Laboratory Diagnostics, University Hospital Split, Split; 4 Clinical Institute of Laboratory Diagnostics, Clinical Hospital Center Rijeka, Rijeka; 5 Department of Medical Informatics, Rijeka University School of Medicine, Rijeka

**Keywords:** negative results, publication bias, research integrity, medical journals

## Abstract

Studies with positive results are greatly more represented in literature than studies with negative results, producing so-called publication bias. This review aims to discuss occurring problems around negative results and to emphasize the importance of reporting negative results. Underreporting of negative results introduces bias into meta-analysis, which consequently misinforms researchers, doctors and policymakers. More resources are potentially wasted on already disputed research that remains unpublished and therefore unavailable to the scientific community. Ethical obligations need to be considered when reporting results of studies on human subjects as people have exposed themselves to risk with the assurance that the study is performed to benefit others. Some studies disprove the common conception that journal editors preferably publish positive findings, which are considered as more citable. Therefore, all stakeholders, but especially researchers, need to be conscious of disseminating negative and positive findings alike.

## Introduction

Studies with successfully proven hypothesis are represented in the literature in a greater amount than studies that “failed” to prove the hypothesis, delivering so-called negative results. It seems that “successful and productive” studies are more interesting, readable and therefore more “valuable” for publishers, editors and readers. This can be derived from the fact that the positive results are more favourably cited in the scientific and medical literature (*1*, *2*). The proportion of positive results in scientific literature increased between 1990/1991 reaching 70.2% and 85.9% in 2007, respectively. On average, yearly increase was 6%, and this effect was constant across most of the disciplines and countries (*3*). Not reporting clinical studies is also evident as merely half of the clinical studies approved by research ethics committee of the University of Freiburg in Germany were published in the form of a full article eight to ten years later, meaning that the other half stayed unpublished and potentially forgotten (*4*).

Moreover, many scientists have produced studies with no proven hypothesis and regarded them as unimportant, unworthy or simply not good enough for publishing. Scientist Peter Dudek illustratively posted in his Tweeter comment from 2013: “If I chronicled all my negative results during my studies, the thesis would have been 20,000 pages instead of 200.”

So what are negative results? There are three cases of negative results:

The study is too small and lacks power. Findings inconclusively suggest no effect.
Despite large enough sample and well-planned study, findings clearly suggest no effect.
Instead of desired outcome study produces the opposite effect entirely (*5*).

The first case is the result of poorly planned study and regardless of the study outcome, those should not be published or interpreted in the context of their limitations. The second case is the genuine case of negative result outcome and such studies are well designed and well executed and therefore deserve to be published. Arguably, the third example is not an actual case of negative results as even though the study does not confirm the tested hypothesis, it is still showing a significant, albeit opposite, effect.

Not submitting and publishing studies brings forth many issues regarding ethics, statistics and finances. The aim of this article is to discuss occurring problems around publishing negative results of well-designed and well-executed studies. We report consequences of underreporting negative results to emphasize why all stakeholders - authors, journal editors and publishers alike, should strive for a more accurate interpretation of scientific results.

## Problems encountered when submitting and publishing negative results

After tedious work of designing a study, recruiting study participants, obtaining necessary finances, ethics committee approval and patients’ informed consents, collecting samples and performing experiments what happens when statistical analysis of results reveals no statistical significance? The scientist who performed the work understands that behind those ‘’negative’’ results is a great amount of effort, time and resources invested and as such, those results have their value and should be published. Unfortunately, there are challenges that our hypothetical scientist is about to face during the process of submitting the work for publishing, challenges that discourage many from publishing their negative results.

## Editors favour studies with positive results

Arguably, many editors prefer to publish positive results, which are considered more intriguing and ultimately are more citable (*1*, *2*, *6*). Multiple analyses showed that papers are more likely to be published, cited and accepted by high-ranking journals if the reported results are positive (*1*, *3*, *7*-*9*).

Once a manuscript is submitted to medical journals, studies have not found statistically significant differences between publishing positive and non-positive results (*6*, *10*-*12*). The editorial preference of positive studies on drug randomized controlled trials (RCT) was also disputed in the analysis of submitted and published RCTs in eight medical journals and a study examining published anaesthesia research suggesting that the reason for publication bias primarily lies with the authors (*11*, *12*).

## Authors favour submitting and citing positive results

Now let us consider a more realistic scenario - our hypothetical scientist works in a clinical laboratory, where his responsibilities also entail assessing and providing a desirable analytical quality of test results, interpreting critical results to clinicians, educating students, dealing with laboratory finances, self-educating and inevitably participating in different research projects. How would our scientist deal with the perceivably difficult task of publishing an article with negative results? It is almost reasonable to think that he would, as many authors confessed, consider the effort of writing up an article to be in vain if the rejection was to be expected and would, therefore, choose to publish work with positive results to maximize his output (*13*).

Some studies state that the publication bias mostly originates when scientists choose not to report their findings as the rejection by the journal is only 6% of all the reasons for non-publishing (*14*, *15*). On the contrary, the main reasons reported by the investigators were lack of time and priority, incomplete study, a study not for publication, manuscript in preparation or under review, unimportant or negative results, low study quality, fear of rejection, rejection and others (*15*).

## Data misinterpretation for obtaining “better suited” results

It has been argued that competition in science contributes to misinterpretation and distortion of results (*7*). In a desire to find a positive outcome of the study, authors can succumb to the pitfalls of focusing on the positive rather than the negative (*16*). Facing non-significant results authors can decide to tweak the hypothesis to better suit their data, also known as HARKing – hypothesizing after the results are known (*17*). HARKing entails meticulous examination or complete disregard of the data that does not fit into the tested hypothesis. In addition, there are numerous reports of scientific misconduct where scientists have completely falsified the published data (*18*).

## Obstruction in the dissemination of research results by the interested parties

Another serious problem that researchers face is the deliberate obstruction of the dissemination of research results by the interested parties, such as sponsors and pharmaceutical companies, whose interest is not to make negative findings publicly available. Industry-sponsored RCTs in neurodegenerative diseases, paediatrics, surgery and cardiovascular medicine were less likely to get published compared to academy sponsored studies (*19*-*22*).

## Consequences of not publishing negative results

Authors often do not think about the aftermath of not publishing negative results and “failed” studies. Logically, the invested money, time and resources go wasted if the study results remain unpublished and unreported. Furthermore, if one scientist has considered a hypothesis was worth exploring, chances are somebody else has had the same or similar idea. Therefore, not publishing a “failed” study can waste other researchers’ time and money in a study that will presumably also produce negative results. Ultimately, this vicious circle leads to personal discouragement and a lot of wastefully spent resources that could have been used to build upon already tested hypothesis.

Clinical trials’ results, especially studies with serious adverse events, often remain unpublished (*23*). Studies, which remain unpublished for reasons other than having negative results, have an unknown and unpredictable effect on the results of meta-analyses (*24*). However, it is clear that a positive bias is introduced when studies with negative results remain unreported, thereby jeopardizing the validity of meta-analysis (*25*, *26*). This is potentially harmful as the false positive outcome of meta-analysis misinforms researchers, doctors, policymakers and greater scientific community, specifically when the wrong conclusions are drawn on the benefit of the treatment.

Furthermore, human subjects have given their informed consent for participating in a study with the assurance that the research is done to benefit others or to contribute to scientific advancement. These participants exposed themselves to risk and therefore, our moral obligation is to publish the results, no matter the outcome of the study (*5*).

Unpublished negative results nourish the interests of those who benefit from these results being hidden. Pharmaceutical companies often do not have the incentive to publish negative results of drug investigations. Alarmingly, 60% of clinical trials with findings of inadequate drug efficacy or safety concerns remained unpublished (*27*). Reporting these results may save time and money for patients and society, as well as prevent possible previously unknown side effects that are usually discovered after new drugs are released (*28*).

## Position of major organizations, funders and journal editors on publishing negative results

The overwhelming issue of publication bias and its effects on the validity of meta-analysis has forced many organizations to tackle this problem by implementing recommendations and mandates. The International Committee of Medical Journal Editors (ICMJE) recommends that journals should require the registration of clinical trials to be eligible for publication. ICMJE believes that there is an ethical obligation to share data generated by such trials: “…to prevent selective publication and selective reporting of research outcomes, and to prevent unnecessary duplication of research effort” (*29*). World Health Organization (WHO) in 2005 published a Statement on Public Disclosure of Clinical Trial Results in which the registration of clinical trials is mandatory before the first study participant is included as well as strict timeframes on reporting the trial results. The Statement also encourages publishing the results of past unreported clinical trials (*30*). This is supported by the Consolidated Standards of Reporting Trials Statement, which includes a requirement to register clinical trials at the time of their inception precisely because of the evident underreporting of clinical trials (*31*). The Declaration of Helsinki clearly states that all contributors, including researchers, authors, sponsors, editors and publishers, are ethically obligated to disseminate the results of research (*32*). Committee on Publication Ethics (COPE) states that studies with negative results should not be excluded in order to support a debate in science (*33*). Publication bias is recognized by research funders that consider publishing negative results should be a priority (*34*).

The effects of publication bias have not gone unnoticed among scientists and clinicians as they reported in an online survey that nearly 70% of researchers were unable to reproduce published results (*35*). Because of a high rate of published results that were found irreproducible and considering that many results of clinical trials have gone unpublished, researchers have organized an All Trials campaign that promotes the publication of currently unpublished clinical trial studies. This campaign urges all stakeholders to implement measures to achieve publication of such results with the aim of obtaining all evidence about the treatment effect (*36*).

There are a number of journals whose scope is to publish negative results in the respective science fields to compensate for evident publication bias (*i.e.,* Journal of Articles in Support of the Null Hypothesis, Journal of Negative Results in BioMedicine, Journal of Pharmaceutical Negative Results, Nature Negative Results section). This, however, can introduce bias in favour of negative results which is counterproductive. Criteria for publishing should be the quality of the study and its power, no matter the outcome of the study. The results obtained from a methodologically well-designed study are trustworthy no matter if they confirm or disprove the null hypothesis.

## Assessing the validity of negative results

With the emphasis on publishing negative results authors, reviewers and editors need to discern true negative results from those obtained from a poorly designed and executed research. Attention should always be paid to study quality and not prioritize the preferable outcome.

The validity of negative results can be assessed like the sensitivity of a diagnostic test (*37*). If using a diagnostic test with low sensitivity, a negative result of the test cannot rule out the disease. Likewise, an effect size and confidence intervals of negative results study should be reported to correctly assess the clinical significance of the results. Discouragingly, only 30% of negative studies published in prominent medical journals have reported studies of power and/or sample size calculations (*37*).

Every time a hypothesis is tested we risk falsely concluding that observed results are not due to chance and that effect exists (type I error; false positive – assessed by alpha statistics) or incorrectly concluding that results are due to chance when in fact the effect is present (type II error; false negative – assessed by beta statistics). Oberhofer and Lennon give emphasis to beta statistics urging the authors to apply the same criteria for detecting a Type II error as a Type I error. Negative results should be interpreted more rigorously to assert that the lack of difference is not due to chance. They state that if we historically accept the 95% confidence that a null hypothesis can be rejected it should also be applied to beta statistics (*38*).

## Conclusions

The studies with findings suggesting no effect or opposite effect are considered negative results (*5*). It has been shown that proportion of positive results in scientific literature in most disciplines has been increasing in past years which entails a reduction in the proportion of negative results (*3*). Consequences of leaving negative findings unreported, apart from an unproductive expenditure of time, motivation and resources, are a positive bias in meta-analysis and drawing erroneous conclusions which ultimately present a serious harm for scientific endeavour (*25*, *26*). Major organizations recommend and encourage reporting results of all clinical trials in the interest of ethical dissemination of studies and drawing unbiased conclusions (*29*-*33*).

*Biochemia Medica* considers all submitted manuscripts irrespective of the positive or negative findings as long as the manuscript is in the scope of the journal and prepared according to the Instructions to authors. The recommendations on the way the results should be reported for all manuscripts regardless of the positive or negative findings are the same and they comply with the recommendations of all leading organizations. Whether the results are negative or positive, it is recommended to report effect size and confidence intervals. Researchers, journal editors and funders need to be conscious of the importance of negative results and report and support disseminating negative and positive findings alike.
